# The role of preoperative immunonutrition on morbidity and immune response after cystectomy: protocol of a multicenter randomized controlled trial (INCyst Trial)

**DOI:** 10.1186/s13063-024-08536-5

**Published:** 2024-10-17

**Authors:** Laurent Derré, François Crettenand, Nuno Grilo, Kevin Stritt, Bernhard Kiss, Thomas Tawadros, Sonia Domingos-Pereira, Beat Roth, Yannick Cerantola, Ilaria Lucca

**Affiliations:** 1 Department of Urology, University Hospital of Lausanne, University of Lausanne, Lausanne, Switzerland; 2 Department of Urology, University Hospital of Bern, University of Bern, Bern, Switzerland; 3 Department of Urology, Hospital of Riviera-Chablais, Rennaz, Switzerland

**Keywords:** Immunonutrition, Bladder cancer, Cystectomy, Infection, Complications, Microbiota, Immune cells

## Abstract

**Introduction:**

Cancer, malnutrition, and surgery negatively impact patient’s immune system. Despite standardized surgical technique and the development of new perioperative care protocols, morbidity after cystectomy remains a serious challenge for urologists. Most common postoperative complications, such as infections and ileus, often lead to longer length of stay and worse survival. The immune system and its interaction with the gut microbiota play a pivotal role in cancer immunosurveillance and in patient’s response to surgical stress. Malnutrition has been identified as an independent and modifiable risk factor for both mortality and morbidity. Immunonutrition (IN) may improve the nutritional status, immunological function, and clinical outcome of surgical patients. Aims of the study are (1) to evaluate the impact of IN on morbidity and mortality at 30 and 90 days after cystectomy and (2) to determine immune and microbiota signature that would predict IN effect.

**Methods:**

This is a randomized, multicentric, controlled, pragmatic, parallel-group comparative study, supported by the Swiss National Science Foundation. A total of 232 patients is planned to be enrolled between April 2023 and June 2026. Three participating centers (Lausanne, Bern, and Riviera-Chablais) have been selected. All patients undergoing elective radical and simple cystectomy will be randomly assigned to receive 7 days of preoperative IN (Oral Impact^®^, Nestlé, Switzerland) versus standard of care (control group) and followed for 90 days after surgery. For the exploratory outcomes, blood, serum, urine, and stool samples will be collected in patients treated at Lausanne. In order to determine the impact of IN on immune fitness, patients enrolled at Lausanne will be vaccinated against influenza and the establishment of the vaccine-specific immune response will be followed. Analysis of the microbiota and expression of argininosuccinate synthetase 1 as potential biomarker will also be performed.

**Discussion and conclusion:**

Strengths of the INCyst study include the randomized, multicenter, prospective design, the large number of patients studied, and the translational investigation. This study will challenge the added value of preoperative IN in patients undergoing cystectomy, assessing the clinical effect of IN on the onset of postoperative morbidity and mortality after cystectomy. Furthermore, it will provide invaluable data on the host immune response and microbiota composition.

**Trial registration:**

ClinicalTrials.gov NCT05726786. Registered on March 9, 2023.

**Supplementary Information:**

The online version contains supplementary material available at 10.1186/s13063-024-08536-5.

## Introduction

Radical cystectomy (RC) with extended bilateral pelvic lymph node dissection (PLND) represents the best available treatment for muscle-invasive BC [1]. Furthermore, this major surgery may be the treatment of choice for non-malignant pathologies, such as interstitial cystitis, painful bladder syndrome, neurogenic bladder, hemorrhagic radiation cystitis, infectious diseases of the bladder, endometriosis, and refractory genitourinary fistulae [2] in patients who have failed previous conservative therapy.

Although cystectomy is considered as an extended major surgery, it has unique features that preclude any outcomes extrapolation from other abdominal surgeries (i.e., colonic resections, gynecologic procedures). These include lymph node resection, longer operative time, significant bleeding, urinary diversion with bowel resulting in metabolic changes, and peritoneal soiling with urine [3].

Despite standardized surgical technique and the development of new perioperative care protocols, cystectomy morbidity remains a serious challenge for urologists. Postoperative complications lead to longer length of stay and worse survival. Infectious complications, including pneumonia, urinary tract infections, surgical site infections, septicemia, and shock, are relatively frequent after cystectomy, rated between 25 and 45% [4, 5]. Patients undergoing cystectomy for a bladder cancer or a benign disease have the similar risk of complications [6]. Antibiotic prophylaxis practices are highly heterogeneous in cystectomy and there is a lack of adherence in guidelines [7].

Careful perioperative risk reduction in patients undergoing major surgery is an evolving key concept to decrease postoperative morbidity rates. Many risk factors, such as repeated radio-chemotherapy regimens, pre-existing co-morbidities, and increased age, can hardly be influenced. Although malnutrition has been identified as an independent risk factor for morbidity and mortality after cystectomy [8–10], there is no recommendation with regard to preoperative screening and/or treatment of malnutrition in guidelines for cystectomy. In most of hospitals where cystectomies are performed, there is no standardized malnutrition screening policy and malnourished patients undergoing surgery are therefore neither identified nor treated.

Another major pathogenic factor leading to postoperative morbidity is the so-called surgical stress response. Nowadays, it has been clarified that surgery induces a complex cascade resulting in inflammatory response, immune suppression, and altered metabolism with hypercatabolism, which altogether lead to impaired wound healing and multi-organ failure. The mediators of this endocrine-metabolic stress response are cytokines, arachidonic acid, nitric oxide, and free oxygen radicals. While the mechanisms mentioned above have been extensively studied, to date, no single intervention has been shown to eliminate postoperative morbidity and mortality. Consequently, immunosurveillance and therefore successful cancer treatment relies on maintaining an unharmed immune system.

Immunonutrition (IN) aims to improve the nutritional status, immunological function, and clinical outcome of cancer patients. When applied to cancer patients treated by radiation or chemotherapy (i.e., not undergoing surgery, thus avoiding surgical stress response), IN has been shown to increase T lymphocyte counts and to decrease inflammatory and oxidative stress response [11]. Many randomized controlled trials and meta-analyses have demonstrated that IN allows for a reduction of complication rate, infections, and length of stay after major abdominal surgery [12]. Unfortunately, very few data are available on the impact of IN on infectious complications after cystectomy [13, 14].

The aims of this study are (1) to evaluate the impact of IN on morbidity and mortality at 30 and 90 days after cystectomy and (2) to determine immune and microbiota signature that would predict IN effect.

## Methods

### Study design

**Fig. 1:**
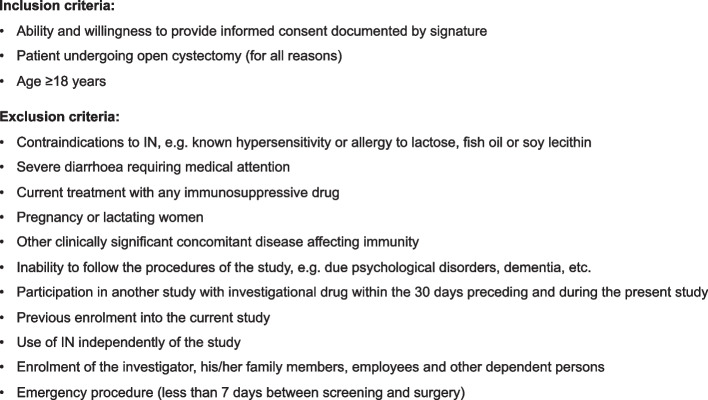
Inclusion and exclusion criteria of the INCyst Trial

The present study (approval #2022–01528 version 5) is designed as a multicenter, prospective, controlled, pragmatic, superiority, parallel-group comparative clinical trial with block randomization stratified by centers (Fig. 1 and Table 1). Patients will be randomly assigned to 2 groups (per center randomization): intervention group (7 days of preoperative oral supplementation with an immune-enhanced oral nutrition (Oral Impact®, Nestlé Nutrition, Switzerland)) or control group (standard of care, no preoperative IN). During an overall study duration of 4 years, a total of 232 patients (116 in each arm) will be included (Table 2 ). Allocation consignment will be generated by a dedicated research nurse at each center, through centralized block randomization via the Interface Web Response System (IWRS) developed within the electronic case report form (eCRF). Equilibrated randomization will be performed on a site-stratified basis to avoid imbalanced randomization between clinical sites where patient recruitment may differ. The allocation sequence is concealed from the investigators enrolling and assessing the patients.

**Table 1:** Trial registration data set according to World Health Organization

Data category	Information
Primary registry and trial identifying number	ClinicalTrials.gov NCT05726786
Date of registration in primary registry	March 9, 2023
Secondary identifying numbers	2022–01528 (Swissethics); SNCTP000005406 (KOFAM)
Source(s) of monetary or material support	Swiss National Science Foundation
Primary sponsor	University Hospital of Lausanne (CHUV)
Secondary sponsor(s)	None
Contact for public queries	Dr. MD. Ilaria Lucca (Ilaria.Lucca@chuv.ch)
Contact for scientific queries	Dr. MD. Ilaria Lucca (Ilaria.Lucca@chuv.ch)
Public title	The impact of immunonutrition on postoperative complications in patients undergoing bladder removal surgery
Scientific title	The role of preoperative immunonutrition on morbidity and immune response after cystectomy: protocol of a multicenter randomized controlled trial (INCyst Trial)
Countries of recruitment	Switzerland
Health condition(s) or problem(s) studied	Determination of infectious complications rate at 30 days after surgery
Intervention(s)	Drug: Oral Impact^®^, Nestlé Health Science, Switzerland
Key inclusion and exclusion criteria	Inclusion criteria: age ≥ 18 years, patient undergoing open cystectomy (for all reasons) Exclusion criteria: contraindications to immunonutrition (e.g., known hypersensitivity or allergy to lactose, fish oil, or soy lecithin), severe diarrhea requiring medical attention, current treatment with any immunosuppressive drug
Study type	Multicenter, prospective, controlled, pragmatic, parallel-group comparative study with block randomization stratified by centers
Date of first enrolment	April 2023
Target sample size	232
Recruitment status	Recruiting
Primary outcome(s)	Determination of the Comprehensive Complication Index (CCI) at 30 and 90 days after surgery Determination of the mortality rate at 30 and 90 days after surgery Determination of the postoperative complication-free survival
Key secondary outcome	Identification of biomarkers predictive of postoperative complications studying the patient’s immune and microbiome signature

Nutritional Risk Status (NRS) Score will be performed in each patient before inclusion. Preoperative carbohydrate loading will be allowed in both groups in case of malnutrition and at discretion of the referent physician. Malnutrition status will not impact the group allocation. This trial will be conducted in accordance with the Good Clinical Practice Guidelines and applicable local regulations. Moreover, we used the SPIRIT reporting guidelines to write the protocol (Supplementary Table 1). The INCyst Trial is supported by the Swiss National Science Foundation (#32003B_207837). The Swiss National Science Foundation is not involved in the design of the study and collection, analysis, and interpretation of data and in writing the manuscript.

**Table 2:** Schedule of enrolment, interventions, and assessments

**Study periods**	**Screening**	**Pre-operative**	**Follow-up**				
**Visit**	**V0**	**V1**	**V2**	**V3**	**V4**	**V5**	**V6**
Time (day)	J-30* /-8	J-1/0	J0+1	J0+5	end of hospitalization	J0+30+/-10	J0+90 +/-20
Informed consent	X						
Demographic characteristics	X						
Medical history	X						
In-/Exclusion criteria	X						
Physical examination	X	X	X	X	X	X	X
Weight		X	X	X	X	X	X
**Laboratory samples (only Lausanne)**							
Blood (50ml/sample)	X	X				X	
Urine (5 to 300 ml/sample)	X	X				X	
Stools	X	X				X	
Arginine level	X	X					
Tissue (ASS1 expression analysis)		X					
Fresh Tissue (oncological patients)		X					
Influenza vaccination	X						
NRS	X						
Eligibility and Randomization	X						
Post-operative infectious complications						X	X
Comprehensive Complication Index (CCI)						X	X
EORTC-QLQ-C30 questionnaire	X					X	X
Peri-operative management					X		

### Clinical outcomes

The primary endpoint is to investigate the infectious complications rate at 30 days after surgery. All type of infectious complication will be considered and graded according to the Clavien’s grade.

Secondary clinical outcome is Comprehensive Complication Index (CCI) evaluated at 30 and 90 days after surgery. Postoperative complications will be graded according to their severity. A validated therapy-oriented, modified Clavien-Dindo classification will be used to asses complication grade [15]: major complications are defined as grade 3–4; grade 5 corresponds to mortality.

Mortality rate at 30 and 90 days after surgery, postoperative complication-free survival, treatment compliance, impact of IN on oncologic and non-oncologic patients, role of IN in patients undergoing neoadjuvant chemotherapy, and quality of life after surgery will also be investigated.

### Exploratory outcomes

For exploratory outcomes, blood, serum, urine, and stool samples will be collected in patients treated at the site of Lausanne. The main goal is to characterize different immune parameters to define a signature that will be predictive of the IN efficacy and that will guide to select patients likely to benefit from IN.

Percentages of different immune cell subsets from the peripheral blood mononuclear cells (PBMCs) will be measured by flow cytometry. We will study the frequency of different subsets of immune cells such as CD3^+^ T cells, regulatory CD4^+^ T cells, natural killer (NK) cells, and myeloid-derived suppressor cells. Furthermore, the expression of immune checkpoints (PD-1, TIM-3, BTLA, CTLA-4, and TIGIT) on T cells from blood will also be performed ex vivo.

To determine the influence of IN on immune response generated in vivo, T-cell specific immune response against influenza will be monitored in the PBMCs before vaccination and after. It is well known that the presence of polyfunctional T cells (i.e., capacity to produce several cytokines at the same time) may be beneficial to the outcome of a disease, especially in cancer [16–18]. Thus, T-cell polyfunctionality assessment may provide a surrogate for potential antitumor immune response. We will stimulate blood T cells from patients in vitro either with anti-CD3 and anti-CD28 antibodies or with a pool of peptides from influenza and measure the production of TNF-α, IFN-γ, IL-2, IL-4, IL-10, IL-17, and CD107a by flow cytometry. We also intend to determine the concentration of inflammatory and suppressive cytokines in serum and urine using the classical Luminex assay, as we did in a previous clinical trial [19].

Furthermore, it is well known now that a disruption of the symbiosis between the microbiota and the immune system, as a result of antibiotic usage or diet alteration, may lead to the development and/or exacerbation of different diseases [20], supporting the fact that IN may impact the gut microbiota. Microbiota diversity and abundance will be thus determined by genomic sequencing and bioinformatics analysis on patient feces. Finally, argininosuccinate synthetase 1 (ASS1) expression will be analyzed by immunohistochemistry in order to evaluate whether its expression may alter the IN efficacy.

Biological material will be appropriately stored in a restricted area only accessible to authorized personnel according to the regulation of the urology biobank of Lausanne University Hospital (https://www.chuv.ch/fr/urologie/uro-home/recherche/axes-de-recherche/biobanque-durologie). No publication or report of any kind will contain patient’s names or any identifying features of single patients. No on-line or written document or communication will refer to participant’s personal detail.

### Patients’ selection, antibiotic regimen, and perioperative protocol

**Fig. 2:**
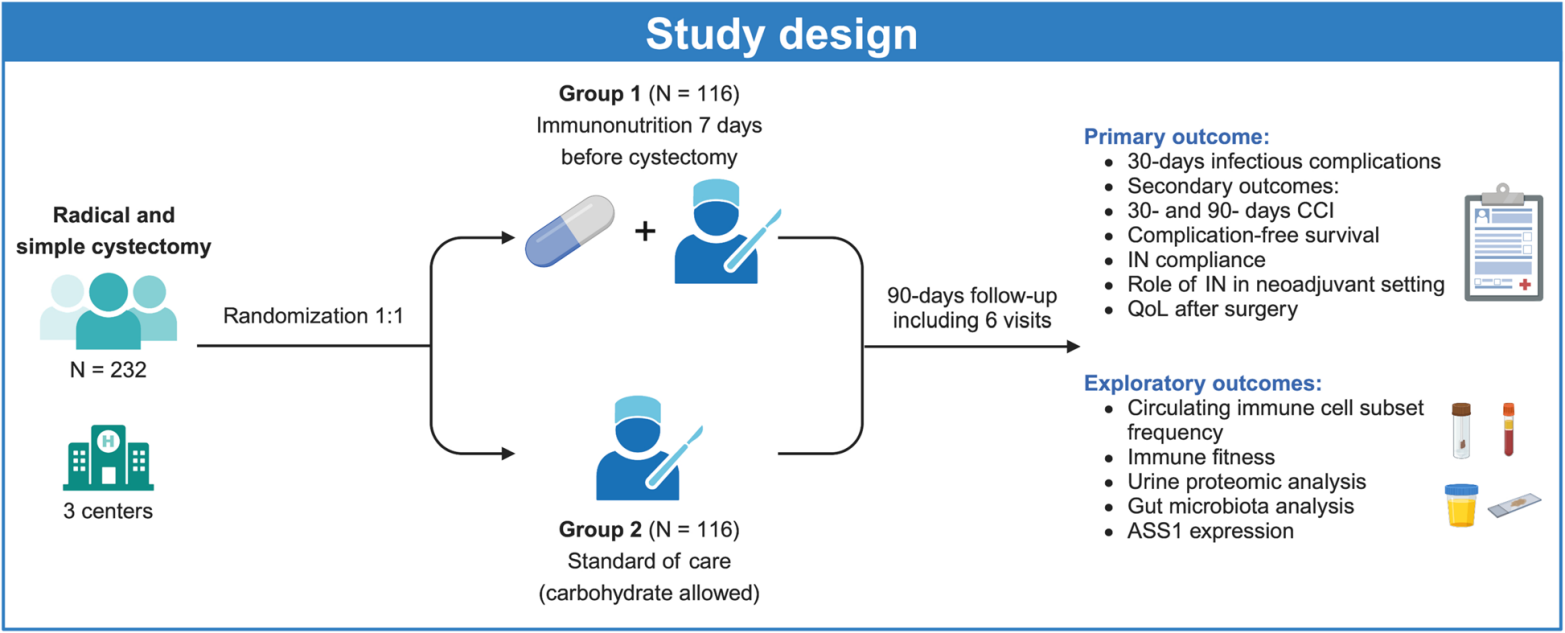
Design of the study. INCyst Trial is a multicenter, prospective, controlled, pragmatic, single-blinded randomized controlled trial assessing the added value of preoperative immunonutrition (IN) on top of standard of care, as well as the clinical effect of IN on the onset of postoperative morbidity after cystectomy. CCI, Comprehensive Comorbidity Index; QoL, quality of life; ASS1, argininosuccinate synthetase 1. Created with BioRender.com

All patients undergoing radical or simple cystectomy (for all reasons) will be included. Inclusion and exclusion criteria are listed in Fig. 2. A urine culture will be performed 10 days before surgery and all patients will receive the same prophylactic antibiotic regimen: cefuroxime iv (single dose) and metronidazole IV (single dose) at the time of surgery for cystectomy with ileostomy/ureterocutaneostomy and cefuroxime iv and metronidazole IV (48 h after surgery) for cystectomy with orthotopic neobladder or heterotopic pouches. Patients will receive oral co-trimoxazole (single dose) at the time of ureteral stent removal. In case of allergies or antibiotic resistances according to the urinary culture, the antibiotic regimen will be adapted, and all changes will be recorded.

Each center will follow the same perioperative protocol according to the ERAS protocol for cystectomy [21]. The surgical procedure will be undertaken in a standardized technique among the three centers. As the robotic access is not routinely performed in Switzerland, only open cystectomies will be included. Bilateral PLND will be performed only in bladder cancer patients as an extended template. The mode of diversion/reconstruction will be left at the surgeon’s discretion but will be nevertheless documented. In case of orthotopic or heterotopic urinary diversion, the Studer technique will be performed.

A dedicated questionnaire (EORTC QLQ-C30) [22] will be distributed to patients assessing patients’ health status. The questionnaire will be discussed with each patient at visit V1 (inclusion), V5 (30 days after surgery), and V6 (90 days after surgery).

### Follow-up of adverse events (AE)

No AE related to the intake of immunonutrition are expected and therefore only 30-day and 90-day postoperative surgical complications will be reported to evaluate primary and secondary endpoints. All serious AEs will be followed until they are resolved or stabilized, including after the end of the study. If immediate safety and protective measures have to be taken during the conduct of the study, the investigator notifies the local ethics committee of these measures, and of the circumstances necessitating them, within 7 days.

### Criteria for discontinuing or modifying interventions

Immunonutrition has been used for more than 15 years and no critical safety issues have been reported. Side effects (i.e., severe diarrhea or nausea requiring medical attention) related to the investigational product will not be considered as a criterion for discontinuation of the intervention.

### Monitoring

The monitoring will be performed by the clinical trial unit of Lausanne, in accordance with ICH GCP E6(R2). The monitor (independent of the research team) will carry out this activity according to a pre-established monitoring plan adapted to the risk of the clinical trial. The monitor will verify that the clinical trial is conducted, and that the data are generated, documented, and reported in accordance with the requirements of the protocol, the GCP, and the applicable regulatory requirements. In concrete terms, an initiation visit, intermediate visits, and a closing visit will be organized on each site by the monitor in accordance with the monitoring plan. At each site, the local principal investigator will ensure that the monitor always has access to the trial data and source documents and will respond to questions and requests for corrections during the monitoring visits.

### Choice of comparators and protocol adherence

Oral Impact^®^ (IN) is an immune-modulating nutritional supplement containing arginine, omega-3 polyunsaturated fatty acids, and ribonucleic acid. Arginine is a key amino acid involved in multiple cellular processes as well as in lymphocyte function and its deficiency results in impaired adaptive immune response. It plays an active role in wound healing, muscle repair (by protein translation), and thrombosis prevention. The present design raises the issue of supplementing patients that are not at nutritional risk with Oral Impact®, resulting in overnutrition with potentially deleterious clinical consequences. However, this hypothesis has not been confirmed by others [23] and the product’s brochure stipulates that the administration of Oral Impact® in the preoperative phase is also indicated in well-nourished patients [24]. Moreover, surgery induces arginine deficiency and many patients with normal preoperative arginine concentrations cannot endogenously satisfy the metabolic demand after surgery.

Patients will be randomly assigned to receive 7 days of preoperative IN, three times a day (Oral Impact^®^) or will not receive oral IN supplements. Carbohydrate loading and protein drinks will be allowed in both groups. In the interventional group, patient’s compliance to the allocated treatment will be recorded by asking the patient how many nutritional supplements she/he has ingested preoperatively (ranging from 0 to 21 bags). Compliant patients will be defined by having drunk at least 14 of the 21 allocated nutritional supplements. To objectively verify and validate these subjective data, serum arginine levels will be assessed at enrolment (between day − 30 and day − 8) and at preoperative admission after intake of the allocated nutrition (day − 1).

Potential effective crossover between intervention groups will be assessed and recorded. As usual with interventions requiring voluntary adherence to a protocol, there is a risk for some participants from the IN arm do not comply with the intervention. Moreover, a mirroring risk exists for participants from the control group to access IN independently of the study protocol, since the product is commercially available. Recording such protocol deviations is mandatory for processing to a second line per-protocol analysis. However, the risks of crossover are very low.

### Protocol amendments

Substantial changes to the study setup and study organization, the protocol, and relevant study documents will be submitted to the local ethics committee for approval before implementation. Under emergency circumstances, deviations from the protocol to protect the rights, safety, and well-being of human subjects may proceed without prior approval of the local ethics committee. Such deviations shall be documented and reported to the ethics committee as soon as possible.

### Roles and responsibilities

The sponsor is the University Hospital of Lausanne (CHUV), which is responsible for providing to the investigator the necessary information to conduct the clinical trial, to ensure proper monitoring of the trial and certifying compliance to ethical bodies and Swiss legislation. The sponsor can be contacted by email (bpr@chuv.ch). The coordinating center is the Department of Urology of CHUV.

A dedicated research nurse, urologist, and data manager in each participating center are responsible for all aspects of local organization, including identifying potential recruits and taking informed consent. A dedicated urologist from the coordinating center (Department of Urology of the CHUV) will be responsible for the grading of postoperative complications. The assessor will be blinded but not the data analyst. The Urology Research Unit will be responsible for the translational research (exploratory outcomes).

### Data management system

The study coordinator will be responsible for entering relevant data from the eCRF or results from the laboratory in Excel data bases. Data generation, transmission, archiving, and analysis of personal data within this study will strictly follow the applicable Swiss legal requirements for data protection. Study data will be entered into the eCRF. The REDCap^®^ software will be used and is validated by the Lausanne University Hospital for the research data management. Prerequisite is the voluntary approval of the subject given by signing the informed consent prior to start of participation in the clinical trial. REDCAP^®^ database runs on a server maintained by the IT Department of the Lausanne University Hospital.

### Data security, access, and back-up

All data will be stored on the Lausanne University Hospital’s secure server. Study data entered into the eCRF are only accessible by authorized persons. Password protection and user right management ensure that only authorized study personal and local authorities (if necessary) will have access to the data during and after the study. Biological material is appropriately stored in a restricted area only accessible to authorized personnel according to the regulation of the urology biobank of Lausanne University Hospital (identification with badge).

### Sample size calculations

The literature review showed a postoperative infectious rate between 25 and 45% among patients treated with cystectomy [4, 5]. Prior clinical data from cystectomy patients at CHUV resulted in an infectious rate at 30 days after surgery of 30%. As showed before, the literature review showed an infection between 25 and 45% among patients treated with cystectomy. Assuming a normal distribution for infectious complications after cystectomy and a common standard deviation in both study arms, desired probabilities of type I error (*α*) of 5% and of type II error (*β*) of 20% (i.e., 80% statistical power) and applying a two-sided test, 110 informative patients per arm (i.e., a total of 220) would be needed to decrease the rate of infectious complications by 17% in the IN group (37% infections at 30 days after surgery in the control group vs 20% in the IN group). Since we anticipate a possible dropout rate of up to 5%, a total of 232 patients are intended for enrolment in the study (116 patients per group). To achieve adequate participant enrolment, three Swiss hospitals participate to this study: University Hospital of Lausanne, University Hospital of Bern, and Hospital of Riviera-Chablais, with an estimated rate of recruitment of 50, 25, and 10 patients per year, respectively.

### Statistical analysis

Ordinal and continuous data will be presented by study group in the form of descriptive statistics, as number of patients, mean, standard deviation, minimum, median, and maximum. Categorical data will be presented by study group using contingency tables with absolute and relative frequencies.

Complications rate will be presented using contingency tables with absolute and relative frequencies, overall and then by study group. The complication rate will be compared between groups using a Chi2 test. Mortality after surgery and postoperative complication-free survival will be analyzed using Kaplan–Meier curves. The study groups will be compared with a log-rank test. Nutritional status will be documented, and subgroup analysis of well-nourished patients randomized to the IN group will be undertaken at the end of the study. No imputations will be done on missing data. So, for longitudinal analysis mixed method analysis will be performed. Non-compliant patients from both arms will be maintained in the study, with no exclusion from the primary intention-to-treat analysis, however withdrawn from the per-protocol analysis. There is no data monitoring committee, owing to very good and well-known safety profiles of the IN and to the short follow-up period.

Other subgroup analyses will be performed (1) to test the effect of neoadjuvant chemotherapy and the risk of malnutrition on the primary outcomes and (2) to test the impact of the ERAS® compliance on the primary and secondary outcomes.

### Insurance

In the event of study-related damage or injuries, the liability of CHUV, as the sponsor, provides compensation, except for claims that arise from misconduct or gross negligence.

### Publications

The results of the projects will be published in peer-reviewed open access scientific journals, in the Clinical Trials register, and presented in scientific congresses. Authorship to publications will be granted according to the rules of the International Committee of Medical Journal Editors (ICMJE). In addition, data of each resulting article with be published on an open data repository (Zenodo). The datasets analyzed during the current study and statistical code are available from the corresponding author on reasonable request, as is the full protocol.

## Conclusion

There is an urgent need of improvement in the perioperative management of cystectomy patients. The INCyst Trial will challenge the added value of preoperative IN in patients undergoing cystectomy, possibly reducing the postoperative morbidity and mortality. It might bring breakthrough changes in the daily urology clinical practice that would also be relevant in other medical disciplines practicing heavy surgeries. Plus, it will provide invaluable data on the host immune response and microbiota composition.

## Supplementary Information

Supplementary Material 1.

## Data Availability

Datasets generated during the study will be kept by the corresponding author and made available upon request.
